# FUNCTIONAL IMPROVEMENT AMONG NURSING HOME RESIDENTS WITH DEMENTIA AFTER SKILLED NURSING FACILITY STAY

**DOI:** 10.1093/geroni/igae098.3987

**Published:** 2024-12-31

**Authors:** Ana Montoya, Pil Park, Megan Jensen, Julie Bynum

**Affiliations:** University of Michigan, Ann Arbor, Michigan, United States; University of Michigan, Ann Arbor, Michigan, United States; University of Michigan, Ann Arbor, Michigan, United States; University of Michigan, Ann Arbor, Michigan, United States

## Abstract

Dementia is associated with decline in physical function that may be exacerbated during a hospitalization. Physical function is potentially modifiable through physical therapy during a short-term skilled nursing facility (SNF) stay after hospitalization. While many nursing home (NH) residents experience functional improvement after a SNF stay, some experience no improvement or even decline. It remains unclear whether long-stay NH residents with dementia benefit from a SNF stay prior to transitioning back to long-term care. This study aimed to determine if NH residents with dementia are as likely to experience functional improvement in activities of daily living (ADL) as those without dementia after a SNF admission. Using Michigan state Minimum Data Set (MDS) data from 2017-2019, we identified long-stay NH residents with Medicare-covered SNF stay in 2018 and qualifying ADL variables (n=2,127 residents with dementia; n=1,595 residents without dementia). We analyzed NH residents’ assessments within 120 days after SNF admission and calculated the Long-Form ADL Self-Performance score. We defined functional improvement as one point decrease in ADL score from the first SNF assessment. Almost two thirds of residents experienced functional improvement (61.2% with dementia vs. 63.3% without dementia; p=0.189). The median time to achieve improvement did not differ between the two groups (16 vs. 14 days, respectively; p=0.063). Cox proportional-hazards models revealed no effect of dementia on functional improvement after incorporating demographic characteristics and clinical indicators (HR=0.94, 95%CI [0.85-1.04]). These results support prior findings that NH residents with dementia achieve similar benefit from a SNF stay compared to those without dementia.

